# DNA methylation of the oxytocin receptor gene predicts neural response to ambiguous social stimuli

**DOI:** 10.3389/fnhum.2012.00280

**Published:** 2012-10-10

**Authors:** Allison Jack, Jessica J. Connelly, James P. Morris

**Affiliations:** ^1^Department of Psychology, University of VirginiaCharlottesville, VA, USA; ^2^Department of Medicine and Division of Cardiovascular Medicine, University of VirginiaCharlottesville, VA, USA; ^3^Robert M. Berne Cardiovascular Research Center, University of VirginiaCharlottesville, VA, USA

**Keywords:** oxytocin receptor gene, functional magnetic resonance imaging (fMRI), DNA methylation, epigenetics, social cognition

## Abstract

Oxytocin and its receptor (OXTR) play an important role in a variety of social perceptual and affiliative processes. Individual variability in social information processing likely has a strong heritable component, and as such, many investigations have established an association between common genetic variants of *OXTR* and variability in the social phenotype. However, to date, these investigations have primarily focused only on changes in the sequence of DNA without considering the role of epigenetic factors. DNA methylation is an epigenetic mechanism by which cells control transcription through modification of chromatin structure. DNA methylation of *OXTR* decreases expression of the gene and high levels of methylation have been associated with autism spectrum disorders (ASD). This link between epigenetic variability and social phenotype allows for the possibility that social processes are under epigenetic control. We hypothesized that the level of DNA methylation of *OXTR* would predict individual variability in social perception. Using the brain's sensitivity to displays of animacy as a neural endophenotype of social perception, we found significant associations between the degree of *OXTR* methylation and brain activity evoked by the perception of animacy. Our results suggest that consideration of DNA methylation may substantially improve our ability to explain individual differences in imaging genetic association studies.

## Introduction

Oxytocin (OXT) is a nonapeptide hormone whose action has been demonstrated to play an important role in a variety of social processes. In addition to its more widely discussed role in sexual behavior (Carter, 1992), maternal behavior (Feldman et al., 2007), and affiliative functions like promoting trust (Kosfeld et al., 2005), pair bonding (Ross and Young, 2009), and encoding of positive social stimuli (Guastella et al., 2008a), OXT also appears to be involved in the detection and processing of basic social stimuli. Notably, intranasal OXT administration increases time spent looking at the eyes when viewing human faces (Guastella et al., 2008b), improves recognition of emotions conveyed by the eyes (Domes et al., 2007), and increases sensitivity to biological motion embedded in a noisy background (Kéri and Benedek, 2009). OXT modulation of these relatively low-level perceptual processes is significant to the extent that these processes appear to facilitate more complex social cognitive tasks, such as understanding the actions and intentions of others (Pelphrey and Morris, 2006).

In humans, the actions of OXT have been reported to be mediated by a receptor that is encoded by the oxytocin receptor gene (*OXTR*) (Kimura et al., 1992). Genetic studies have shown that two common single nucleotide polymorphism (SNP) variants in the *OXTR* gene are associated with individual variability in social behavior. The first variant, rs2254298 (G>A), is associated with autism spectrum disorders (ASD) (Jacob et al., 2007) and unipolar depression (Costa et al., 2009). The second variant, rs53676 (G > A), is associated with decreases in psychological resources such as optimism and self-esteem (Saphire-Bernstein et al., 2011), non-verbal intelligence (Lucht et al., 2009), behavioral and dispositional empathy (Rodrigues et al., 2009), positive affect (Lucht et al., 2009), and parental sensitivity (Bakermans-Kranenburg and van IJzendoorn, 2008). This SNP is also associated with structural differences in oxytonergic brain regions including amygdala and hypothalamus, as well as functional differences during emotional face processing (Tost et al., 2010). Although it is possible to draw associations between genotype and phenotype, the biological mechanisms by which these SNPs impact gene transcription is poorly understood. Moreover, the SNP approach forces us to dichotomize our groups by allele type, while ignoring significant variability within group. Such limitations may be addressed, at least in part, by influential new models of human disease that suggest that expression of risk-related genes could be controlled by epigenetic processes (Bjornsson et al., 2004). Consequently, we have begun considering the impact of epigenetic modifications of *OXTR* on social phenotypes.

Methylation of 5′-Cytosine-phosphate-Guanine-3′ (CpG) dinucleotide pairs in DNA is one type of epigenetic modification that could be associated with variability in behavioral phenotype and neural endophenotype. DNA methylation plays a role in differentiation and development, is commonly associated with transcriptional silencing [in some cases heritable (Kaminsky et al., 2009)], and more recent evidence indicates its involvement in activation of transcription (Lister et al., 2009; as reviewed in Suzuki and Bird, 2008). While tissue-specific variation in DNA methylation has been observed, recent work indicates that, on the whole, methylation patterns are relatively conserved across tissue types within individuals (Byun et al., 2009). Moreover, in some cases, epigenetic modifications assessed peripherally have been demonstrated to serve as useful predictors of the same modifications in the phenotypically relevant tissue such as the brain (Kaminsky et al., 2011). Critically for the current investigation, peripheral indicators of *OXTR* methylation have previously been demonstrated to serve as a valid index of both phenotype and endophenotype in ASD, a disorder impacting social perception and behavior. Gregory et al. demonstrated an association between methylation of CpG site −934 (hg19:chr3:8,810,807-8,810,808) in *OXTR* and the presence of ASD in DNA derived from the blood and from post-mortem brain tissue (Gregory et al., 2009). Of note, increased methylation in this site was linked to decreased *OXTR* transcription in temporal cortex, specifically Brodmann's area 41/42. This brain region is adjacent to the temporal parietal junction and near the anterior portion of the superior temporal sulcus (STS), which has been strongly implicated in social cognition by virtue of its role in biological motion perception (Allison et al., 2000; Pelphrey and Morris, 2006) and mentalizing (Frith and Frith, 2003; Gallagher and Frith, 2003) functions. Given the evidence suggesting OXT's role in social perception in typical adults and *OXTR* methylation related transcription differences in temporal cortex of persons with ASD, we hypothesized that *OXTR* methylation would be particularly predictive of functional differences near the temporal parietal junction.

To assess this prediction, we utilized a classic social perception task first developed by Heider and Simmel (1944) and later adapted by Castelli et al. (2000) in which simple geometric shapes either move and interact in ways that imply animacy [the Animate condition (ANIM)] or display an identical amount of random (RAND) movement (the RAND condition). In general, although participants are given no further instructions than to watch the stimuli, this task appears to elicit not just perception of animacy, but also attribution of intentionality and social-emotional content to the interactions between the geometric shapes (Castelli et al., 2000; Scholl and Tremoulet, 2000). This task reliably recruits a network of brain structures believed to be important for social perception and mentalizing abilities, including the temporal parietal junction (Castelli et al., 2000; Ross and Olson, 2010). Additionally, the degree to which areas that are involved in mentalizing processes are activated by these displays is dependent upon the degree to which participants attend to the intentional, contingent relationship relative to mechanical trajectories of the shapes (Blakemore et al., 2003). In the current experiment, participants viewed these stimuli while functional magnetic resonance imaging (fMRI) data were collected. Participants also provided blood from which DNA methylation level of *OXTR* site −934 was determined.

## Methods

### Participants

Forty-three healthy volunteers were recruited from the Charlottesville area. All individuals gave written informed consent for a protocol approved by the University of Virginia Institutional Review Board (Protocol 15051; Principal Investigator, Jessica J. Connelly). One individual was excluded due to difficulties with anatomical/functional registration. Data from 42 healthy adults (23 men), aged 18–30 years (*M* = 21.9 years), with normal or corrected-to-normal vision and no history of neurological or psychiatric illness were included in the final analysis. The self-reported racial breakdown of participating individuals was as follows: 67% Caucasian (*n* = 28), 17% Asian (*n* = 7), 9% Black, (*n* = 4), and 7% of mixed origin (*n* = 3).

### Blood collection and DNA extraction

Venipuncture was performed at the General Clinical Research Center at the University of Virginia. Eight milliliters of blood were collected in mononuclear cell separation tubes (BD Vacutainer CPT with sodium citrate, BD Biosciences, Franklin Lanes, NJ) from each subject. Upon collection, blood samples were immediately spun at 1800 RCF for 20 min to separate the mononuclear cell fraction per product protocol. The mononuclear cells were then lysed and DNA was extracted using the reagents supplied in the Gentra Puregene Blood Kit (Qiagen, Valencia, CA). DNA was stored at −20°C prior to further analysis.

### Epigenotyping procedures

Two hundred nanogram of DNA extracted from peripheral blood mononuclear cells was subject to bisulfite treatment (Kit MECOV50, Invitrogen, Carlsbad, CA). This converts all non-methylated cytosines in the genome to uracil and allows for the downstream detection of methylated cytosines by sequencing. Twenty nanograms of bisulfite converted DNA was used as a template for PCR using a Pyromark PCR kit (Qiagen, Valencia, CA) and 0.2 uM primers TSL101F (5′-TTGAGTTTTGGATTTAGATAATTAAGGATT-3′) and TSL101R (5′-biotin-AATAAAATACCTCCCACTCCTTATTCCTAA-3′). Samples were amplified in triplicate on three identical PCR machines (C1000 Thermal Cycler, Biorad, Hercules, CA.) The following cycling conditions [Step 1: (95°C/15 min)/1 cycle, Step 2: (94°C/30 s, 54°C/30 s, 72°C/30 s)/50 cycles, Step 3: (72°C/10 min)/1 cycle, Step 4: 4°C hold] were used for amplification of the fragment. This amplifies a region on the coding strand of the OXTR gene that contains site −934 (hg19, chr3:8,810,729-8,810,845). PCR conditions were determined using a set of standards for site −934 at 0, 25, 50, 75, and 100% methylated (theoretical versus experimental Pearson's correlation of 0.9966, *p* = 0.002). Successful PCR amplification of a single fragment that runs at 116 bp was confirmed using agarose gel electrophoresis for each sample and replicate. Underlined nucleotides in primer set indicate insertion of an A or C nucleotide at a variable position (C/T) due to a CpG site within the primer. All samples were amplified in triplicate and randomized for pyrosequencing to account for plate and run variability. On average, samples deviated from the mean ±1.7%. Pyrosequencing was performed using primer TSL101S (5′-AGAAGTTATTTTATAATTTTT-3′) on a Pyromark Q24 using PyroMark Gold Q24 Reagents (Qiagen, Valencia, CA) per the manufacturer's protocol. Epigenotypes reported are an average of three replicates.

### Animations

Sixteen (ANIM) animations and 16 RAND animations were presented to subjects during scanning. All animations had a black background with three white geometric shapes—a triangle, a diamond and a circle. There was a large white square in the middle of the screen. Each sequence lasted approximately 16 s. ANIM animations involved goal-directed behavior such as chasing one another, dancing with one another, and hiding within the square. RAND animations showed the shapes bouncing around the screen along straight paths, like billiard balls, with the same average speed as the objects in the ANIM animations. While the types of movement differed, the overall amount of motion was kept as similar as possible.

### Experimental design

Participants were instructed to simply observe the shapes as they moved along the screen. ANIM and RAND animations were presented in an alternating block design. The same background and shapes appeared across the entire run and the only thing that changed was the movement characteristics that defined ANIM and RAND trials. Participants were scanned over two runs and viewed eight unique ANIM animations and eight RAND animations per run.

### Imaging

Scanning was performed on a Siemens 3 Tesla MAGNETOM Trio high speed imaging device equipped with a 12-chanell head-coil. Participant head movement was minimized using cushioned head stabilizers. 176 high-resolution weighted images were acquired using Siemens' magnetization-prepared rapid-acquired gradient echos (MPRAGE) pulse sequence (TR, 1900 ms; TE, 2/53; FOV, 250 mm; voxel size, 1 mm × 1 mm × 1 mm) and used for coregistration with functional data. Whole brain functional images were acquired using a T2^*^ weighted echo planar (EPI) sequence sensitive to BOLD contrast (TR, 2000 ms; TE, 40 ms; voxel size, 3.0 × 3.0 × 4.2 mm; flip angle = 90°). Twenty-eight transverse slices were acquired, with runs consisting of the acquisition of 125 successive brain volumes.

### fMRI data analysis

#### Preprocessing

Motion was detected by center of mass measurements implemented using automated scripts developed for quality assurance purposes and packaged with the BXH/XCEDE suite of tools, available through the Bioinformatics Information Research Network (BIRN). The following pre-statistics processing was then applied: motion correction using MCFLIRT (Jenkinson et al., 2002); non-brain removal using BET (Smith, 2002); spatial smoothing using a Gaussian kernel of FWHM 5 mm; grand-mean intensity normalization of the entire 4D dataset by a single multiplicative factor; highpass temporal filtering (Gaussian-weighted least-squares straight line fitting, with sigma = 50.0 s). Registration to the Montreal Neurologic Institute (MNI) Template standard space image was carried out using fMRIB's software library linear registration tool (FLIRT) (Jenkinson and Smith, 2001; Jenkinson et al., 2002).

#### Main effects

fMRI data processing was carried out using fMRI expert analysis tool (FEAT) Version 5.98, part of fMRIB's software library (FSL, www.fmrib.ox.ac.uk/fsl). At first level, time-series statistical analysis was carried out using fMRIB's software library improved linear model (FILM) with local autocorrelation correction (Woolrich et al., 2001). The timecourses of ANIM and RAND stimulus presentations were included as individual regressors in three-column format, each convolved with a gamma hemodynamic response function and with temporal filtering applied and a temporal derivative added. An ANIM > RAND contrast was conducted, and the contrast of parameter estimates (COPE) from this analysis for each individual was then entered into higher-level analysis. Higher-level analysis of the data was conducted with a mixed-effects approach using fMRIB's local analysis of mixed effects (FLAME) stage 1 (Beckmann et al., 2003; Woolrich et al., 2004; Mark, 2008), which allows for the carrying up of first-level fixed effects variances to the higher-level analysis and forces the RAND effects variance to be non-negative in order to provide a better estimate of the mixed effects variance. Two regressors were included at higher level: a group mean regressor, used to identify brain regions for which there was a main effect of the ANIM > RAND task contrast; and a regressor including percentage *OXTR* methylation (de-meaned) for each participant, used to identify regions in which ANIM > RAND activity was significantly associated with *OXTR* methylation. A whole-brain analysis was conducted; *Z* (Gaussianised *T*/*F*) statistic images were thresholded using clusters determined by *Z* > 2.3 and a (corrected) cluster significance threshold of *p* < 0.05 (Worsley, 2001). To probe the direction of effects related to *OXTR* methylation, significant clusters associated with the *OXTR* regressor were registered to each participant's native space and average ANIM > RAND values for each individual were extracted from these ROIs.

## Results

DNA methylation of *OXTR* site −934 ranged from 29 to 61% methylated in the 42 healthy adult subjects sampled.

Regions demonstrating significantly greater activity in the ANIM versus RAND condition, were generally consistent with previous literature (Castelli et al., 2000; Schultz et al., 2003): the posterior STS bilaterally, the right precentral gyrus, the left inferior frontal gyrus, the dorsomedial prefrontal cortex, and the anterior intraparietal sulcus (see Table 1).

**Table 1:** **MNI coordinates and response intensity of brain regions displaying a significant response to Animate > Random stimuli *p* < 0.05 (corrected)**.

**Anatomical region**	**Hem**	***x***	***y***	***z***	***Z***	***k***
Posterior superior temporal sulcus	R	50	−66	−8	8.65	13,033
	L	−48	−74	−8	8.82	5671
Precentral gyrus	R	48	8	34	6.21	4976
Inferior frontal gyrus (BA 45)	L	−50	26	14	5.57	2610
Dorsomedial prefrontal cortex	−	2	62	22	4.52	1365
Anterior intraparietal sulcus	L	−38	−38	44	4.74	1307

Note: MNI coordinates are reported. Hem, hemisphere; L, left; R, right; Z, z-statistic; k, cluster size (in voxels).

**Figure 1:**
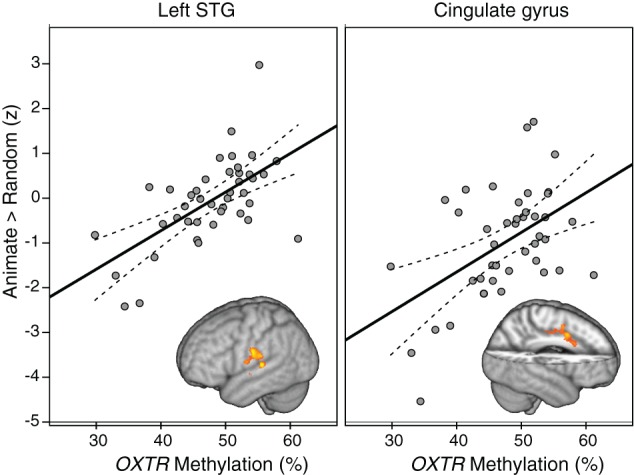
**Regions demonstrating a significant association between *OXTR* methylation and Animate > Random activity.** Clusters from whole-brain analysis are depicted in MNI space and neurological orientation. Average *z*-statistic values from the contrast of parameter estimates (COPE) in the left STG and dACC are plotted against percent *OXTR* methylation for each participant, with dashed lines indicating 95% confidence interval (CI) around fit line.

Whole-brain analysis indicated that degree of *OXTR* methylation was significantly associated with BOLD activity in the ANIM > RAND contrast in two clusters. The first cluster extended from the superior temporal gyrus into supramarginal gyrus at the temporal parietal junction. The second significant cluster was in dorsal anterior cingulate cortex (dACC; Table 2; Figure 1). In both cases, individuals with higher levels of *OXTR* methylation demonstrated greater activity in these regions.

**Table 2:** **MNI coordinates and response intensity of cluster peaks identified in which brain response for the Animate > Random contrast varied significantly as a function of *OXTR* methylation *p* < 0.05 (corrected)**.

**Anatomical region**	**Hem**	***x***	***y***	***z***	***Z***	***k***
Superior temporal gyrus/supramarginal gyrus	L	−64	−24	12	4.20	713
Dorsal anterior cingulate cortex	R	12	6	40	3.65	429

Note: Hem, hemisphere; L, left; R, right; Z, z-statistic; k, cluster size (in voxels).

There have been documented differences in global levels of DNA methylation as a function of ethnicity and gender (Zhang et al., 2011). In order to account for this, we performed a subset analysis of our Caucasian sample, which represents our largest subgroup, and an analysis by gender in our overall dataset. When data were analyzed in the Caucasians only subset (*n* = 28), the overall pattern of effects for *OXTR* methylation remained the same, with activity in both the STG [*r*_(28)_ = 0.72, *p* < 0.001] and the dACC [*r*_(28)_ = 0.48, *p* = 0.010] positively correlated with methylation. With regards to potential gender differences, men and women did not differ on degree of methylation in our sample, nor did they significantly differ in their response to the ANIM > RAND contrast in whole-brain analysis. When controlling for gender, *OXTR* methylation continued to be positively associated with activity in the STG [*r*_(39)_ = 0.54, *p* < 0.001] and dACC [*r*_(39)_ = 0.41, *p* = 0.007] in the full sample. Finally, there was no interaction between gender and *OXTR* methylation in predicting ANIM > RAND response within those clusters we had previously identified as showing an effect of methylation (i.e., dACC, STG).

## Discussion

This study demonstrates that DNA methylation of *OXTR* is associated with individual variability in brain regions supporting social perception. Prior studies have established a link between genetic variability of *OXTR* and phenotypic variability in healthy and disordered populations, as well as variability in brain structure and function. Here, we demonstrate that epigenetic modifications of *OXTR* also impact phenotypic variability and that we may assess epigenetic variability via peripheral blood in a healthy sample of adults. Our results represent an important advance for future models of gene/brain/behavior relationships and provide potential clues to mechanisms underlying social perception deficits in developmental and psychiatric disorders.

We observed a significant relationship between degree of *OXTR* methylation and brain activity evoked by perception of animacy in two brain regions known to play a role in social perception. Specifically, higher levels of methylation were associated with greater activity in temporal parietal junction and dACC. Much of the existing literature on temporal parietal junction has focused on the perception of biological motion cues, attributions of intentions, and mentalizing behaviors. These perceptual skills, referred here collectively as social perception, are sensitive to intranasal administration of OXT. Here we found a relationship between DNA methylation of *OXTR* and BOLD response in a cluster of voxels that span from the left superior temporal gyrus into postcentral and supramarginal gryrus. This region has previously been identified as being sensitive to the degree to which subjects attend to contingency between moving shapes in ANIM displays (Blakemore et al., 2003). Moreover, activity in the adjacent left STS region is sensitive to attributions of causality of biological motion (Morris et al., 2008). Our results indicate that *OXTR* methylation may impact the degree to which individuals are sensitive to displays of ANIM motion, perhaps indicating differing degrees of attention to the cause of motion or social contingencies between shapes relative to mechanical aspects of motion. This perceptual sensitivity, or style, could be indicative of a social style that varies within the population and is compromised in developmental disorders such as ASD.

The relationship between OXT, dACC, and social and emotional behavior is complex. Despite direct anatomical connections between dACC and the limbic system, most traditional neurocognitive models posit a role for dACC in executive functions such as modulation of attention and cognitive control (Bush et al., 2000). However, more recent evidence suggests that this region plays a critical role in social and affective appraisals of motivationally salient stimuli (Etkin et al., 2011). Moreover, abnormal function of dACC has been associated with emotional and social perceptual deficits in anxiety disorders (McClure et al., 2007) and ASD (Dichter et al., 2009). Structural variability of dACC has also been associated with two common genetic variants found within *OXTR.* Carriers of the risk allele of *OXTR* SNP rs2254298 have increased dACC volume relative to non-risk allele carriers (Furman et al., 2011). A second SNP, rs53576, is associated with volume differences in hypothalamus and the degree to which dACC is structurally coupled with hypothalamus. Specifically, risk allele carriers show increases in hypothalamus volume and the degree of connectivity between hypothalamus and dACC (Tost et al., 2010). The sample size of the current study does not allow for appropriate measurements of interactions between polymorphic variability and *OXTR* methylation. Thorough examination of SNP × DNA methylation interactions will be critical in future neurogenetic association studies and may provide further clues to unravel the complex interplay of genomic association and social behavior.

The direction of the relationship between *OXTR* methylation and brain activity might seem counterintuitive. If higher degrees of DNA methylation lead to decreased transcription of *OXTR* in the brain (Kusui et al., 2001; Gregory et al., 2009) one might expect processes mediated by OXT to be more perturbed in individuals high in DNA methylation. Here, we report the opposite—specifically, we report a strong positive relationship between *OXTR* methylation and activity associated with perception of animacy. We believe there are at least two plausible explanations for the pattern of findings reported here. First, increased activity may indicate more resource-intensive processing. Activity in pSTS, for example, has previously been found to increase in situations in which more elaborated processing of moving social stimuli is required, such as when a character's actions are incongruent with expectations (Pelphrey et al., 2003, 2004). Similarly, given the role of dACC in resolving ambiguity in expressed emotion (Nomura et al., 2003), a main effect of methylation in this region could indicate that persons with higher levels of *OXTR* methylation perceive the presented ANIM interactions as more ambiguous, and consequently recruit dACC more heavily in an attempt to process these stimuli. Second, the range of methylation we detected (29–61%) does not compromise the entire theoretical range (0–100%); therefore, the positive correlation we note here may only be applicable to typically functioning individuals. The pattern of association could change such that extremely high levels of methylation may predict *lower* activity in brain areas associated with social perception.

The current approach relates DNA methylation evaluated from peripheral blood with brain activity assessed by fMRI. Implicit in this approach is the notion that DNA methylation of *OXTR* assessed from peripheral blood is similar in the brain, is related to individual variability in *OXTR* expression in the brain, and that these expression differences impact brain function. The relationship between *OXTR* DNA methylation at site −934 from human peripheral blood and that found in the same individual's brain tissue is currently unknown. However, there exists the possibility that this CpG site is inherited or established early in development and relatively stable. While traditional models of epigenetic inheritance proposed erasure of DNA methylation in non-imprinted regions between generations, contemporary reports in model systems and humans suggest that in some cases this epigenetic modification may survive reprogramming (Daxinger and Whitelaw, 2012; Labrie et al., 2012). Twin studies have indicated that methylation level at specific CpG sites are more commonly shared by twins when compared to unrelated individuals, are correlated between tissues and can be stable across the life span (Kaminsky et al., 2009; Bell et al., 2012).

The current study has two important limitations with regard to our sample. First, our conclusions are restricted to psychologically and physically healthy adults. It is possible that the pattern we note here is only applicable to individuals within the normal range; in other words, past a certain degree of *OXTR* methylation, higher levels of methylation may no longer predict greater brain activity in regions described here. As increasing effort leads to diminishing returns, individuals with pathological levels of methylation may either become less able to attribute animacy to these displays or may recruit different brain systems to process them. Second, we are unable to infer how the relationship between *OXTR* methylation and brain activity associated with social perception may predict differences in overt social behavior. It's possible that in healthy populations, the relationship between epigenetic variability and endophenotypes may be unrelated to social behavior. Alternatively, we may find that consideration of epigenetic variability will give us a new tool to understand the contribution of genetic variability to individual differences in healthy and disordered populations.

### Conflict of interest statement

The authors declare that the research was conducted in the absence of any commercial or financial relationships that could be construed as a potential conflict of interest.
